# Change of Characterization and Film Morphology Based on Acrylic Pressure Sensitive Adhesives by Hydrophilic Derivative Ratio

**DOI:** 10.3390/polym12071504

**Published:** 2020-07-07

**Authors:** Woong Cheol Seok, Jong Tae Leem, Ju Hui Kang, Young Jun Kim, Sangkug Lee, Ho Jun Song

**Affiliations:** 1 Green and Sustainable Materials R&D Department/Research Institute of Clean Manufacturing System, Korea Institute of Industrial Technology, 89 Yangdaegiro-gil, Ipjang-myeon, Seobuk-gu, Cheonan-si, Chungcheongnam-do 331-822, Korea; vmfosel07@kitech.re.kr (W.C.S.); dla700@kitech.re.kr (J.T.L.); juri312@kirtech.re.kr (J.H.K.); skdlee@kitech.re.kr (S.L.); 2 School of Chemical Engineering, Sungkyunkwan University, 2066 Seobu-ro Jangan-gu, Suwon-si, Gyeonggi-do 440-746, Korea; youngkim@skku.edu

**Keywords:** acrylic pressure sensitive adhesive, photo-polymerization, morphology, rheology

## Abstract

Hydrophilic acrylic pressure-sensitive adhesives (PSAs) were synthesized by controlling the contents of 2-ethylhexyl acrylate (EHA), isobornyl acrylate (IBOA), and 2-hydroxyethyl acrylate (HEA); especially, the characteristic change of the HEA content was analyzed. Surface contact angle of acrylic PSA film decreased from 77.87° to 70.23° in the case of Acryl-2 to Acryl-8 (below HEA 10 wt %). However, the surface contact angle of Acryl-10 to Acryl-40 (HEA 10 wt % to 40 wt %) increased up to 92.29°, indicating hydrophobicity. All acrylic PSA films showed high adhesive force above 1800 gf/25 mm. According to X-ray diffraction (XRD) measurement, hydrophilic acrylic PSAs exhibited amorphous property and it was confirmed that the morphology of acrylic PSA film was significantly affected by the flexibility of the polymer chain and the strength of hydrogen bonding. The affinity with hydrophilic materials for acrylic PSA films was evaluated by T-type peel test, confirming that the affinity with hydrophilic materials is determined by the hydrophilicity of the acrylic PSA film. The synthesized acrylic PSA film is non-toxic regardless of the hydrophilicity.

## 1. Introduction

Pressure-sensitive adhesives (PSAs) are representative viscoelastic materials with various applications such as automobiles, liquid crystal display (LCD)/organic light emitting diode (OLED) displays, medical tape/dressing band, etc. [1,2,3,4]. These PSAs have the following advantages: (1) they are firmly attached to a general substrate or skin immediately under light pressure and in a short time; (2) the attach and detach process can be repeated after a period of time; (3) they are easier to use than quick reacting two-component type adhesives; and (4) it is easier to control the PSA properties through processes such as monomer composition and curing.

These PSAs are classified into various types based on the polymers used, such as adhesives based on acrylate [5], rubber [6], silicone [7], polyurethane [8], etc. Among them, PSAs based on acrylic polymers (acrylic PSAs) have made tremendous advances for industrial fields and have been suggested in delicate sciences. They are widely used in various industrial areas because of advantages such as easy access of molecular structure, as well as can be designed and synthesized for various applications. Acrylic PSAs have excellent advantages in transparency, weather resistance, and heat resistance, and they consist of a soft segment, a hard segment, and a functional segment. The soft segment affects the initial adhesive strength and flexibility of the adhesive and the hard segment affects the cohesive property of the adhesive. The functional segment controls the crosslinking point of the acrylic PSAs to control the crosslinking density for determining properties [9]. The combination of these monomers can exhibit various properties and the have been applied in diverse fields, from general household materials to many high-tech industries, such as automotive, aviation, and information technology (IT) [10].

The synthesis of acrylic PSAs can be divided into two types: solvent and solvent-free. Solvent type polymerization uses an organic solvent when synthesizing the acrylic PSAs, and it is common to conduct a reaction at a higher temperature of 70–80 °C for the activation of the initiators. This method has advantages in synthesis and process control as it is easy to control reaction, heat, and viscosity because of the solvent [11,12,13,14,15,16]. However, since the reaction conversion rate of the monomer is less than 100%, the unreacted monomers remain after the reaction. In addition, the organic solvent must be removed before use. Recently, solvent-free polymerization without using organic solvent has attracted attention as an alternative for environmental pollution reduction measures, conforming to environmental regulations. Typical solvent-free polymerizations include hot-melt type, photo-curable type, and aqueous type. Among them, photo-curable polymerization is 100% solid because it is a bulk-polymerization consisting of a monomer and initiator only; thus, it has low environmental pollution, fast curing speed, and simple process. Therefore, it is more advantageous than conventional thermal polymerization in terms of facility, productivity, and energy reduction. Besides, a low-temperature curing process is possible because of UV curing, and, since the unreacted monomer does not exist after the reaction, it can be more widely applied in industry than the solvent-type polymerization [5,17,18].

To apply acrylic PSAs in the medical field, they must be non-toxic first, and compatible with medical materials such as drugs or other biocompatible materials. The compatibility with medical materials is determined by the polarity of acrylic PSAs, and different types of acrylic PSAs can be designed according to the purpose of the adhesive and the medical materials. The polarity of the acrylic PSAs can be controlled in polar or non-polar form depending on the functional groups introduced to the backbone or side of the adhesive, which can be applied as patches in various forms depending on the controlled polarity [19,20,21,22].

Many drugs used in medical patches exhibit lipophilic properties; therefore, many studies have been conducted on non-polar hydrophobic acrylic PSAs [13,20,23,24]. However, with the development of the medical device industry, polar hydrophilic materials are being developed. Accordingly, the necessity of hydrophilic PSAs is also gradually increasing. However, studies on hydrophilic PSA have not yet been conducted. Recent studies have attempted to improve the hydrophilicity of the adhesive by introducing polyethylene glycol (PEG) (a hydrophilic functional group) into styrene–isoprene–styrene (SIS) (a hot-melt adhesive having hydrophobic properties) onto the backbone of the adhesive or blend [25,26]. However, these methods have a disadvantage in that their application is limited, because they can only be applied in the case of a hot-melt type or a solvent type of acrylic PSA.

In this study, we synthesized acrylic PSAs through photo-polymerization and controlled their hydrophilic properties using the content of a hydrophilic acrylate, 2-hydroxyethyl acrylate (HEA). In addition, the change in morphology and rheology of the synthesized hydrophilic acrylic PSAs were investigated considering the hydrophilic functional group according to the properties of polymer chains, chain orientation, and secondary bonding between chains. We investigated a simple method for evaluating affinity using the peel strength of hydrophilic acrylic PSAs and hydrophilic materials, polyvinyl alcohol (PVA) and hyaluronic acid (HA), and confirmed the potential for medical applications of hydrophilic acrylic PSAs through cytotoxicity and skin irritation tests.

## 2. Materials and Methods

### 2.1. Materials

2-Ethylhexyl acrylate (EHA, 98%), isobornyl acrylate (IBOA), 2-hydroxyethyl acrylate (HEA, 96%), PVA, HA, and isophorone diisocyanate (IPDI, 98%) were purchased from Sigma Aldrich (Seoul, Korea). Irgacure 184 and darocur TPO (photoinitiators) were purchased from Shinyoung Rad Chem (Seoul, Korea). 1-dodecanethiol (98%), a chain transfer agent, was purchased from Alfa Aesar. SA3000, a commercial acrylic PSA with hydrophobicity (Mw: 700,000–800,000 g/mol, contact angle: 95.19°), was purchased from Samwha Co., (Seoul, Korea) All materials were used without further purification.

### 2.2. Synthesis of Hydrophilic Acrylic Pre-Polymers

The hydrophilic acrylic pre-polymers were synthesized by photo-polymerization under UV irradiation. 2-Ethylhexyl acrylate (EHA), isobornyl acrylate (IBOA), and 2-hydroxyethyl acrylate (HEA) were added to three-neck round bottom flasks; photoinitiator (irgacure 184, 1.5 wt %) and chain transfer agent (1-dodecanethiol, 0.01 wt %) were added in the same ratio, as shown in Table 1. The mixtures were stirred at 25 °C with a mechanical stirrer and degassed by bubbling with nitrogen gas for 30 min. After 30 min, the mixtures with acrylate were polymerized by UV irradiation for 10 min through a UV lamp (black light, 10 W) installed outside the flask under nitrogen atmosphere. Further, hydrophilic acrylic pre-polymers were fabricated by mixing the photoinitiator (darocur TPO, 1 wt %) with synthesized acrylic polymers after reaction.

### 2.3. Fabrication of Hydrophilic Acrylic PSA Films

The synthesized acrylic pre-polymers were uniformly applied on the polyester (PET) film with a roller-type coater to a thickness of 100 um and covered with the PET release liner. Thereafter, the coated pre-polymers were cured with UV irradiation (2000 mJ) using UV curing equipment (DMH-1200, DTX, Seoul, Korea) to produce a hydrophilic acrylic PSA films.

### 2.4. Characterization of Hydrophilic Acrylic Pre-Polymers and Acrylic PSA Films

The molecular weight of synthesized acrylic pre-polymers was measured using gel permeation chromatography (GPC P-4000, Futecs, Daejeon, Korea) with a refractive index (RI) detector. The column was used at 40 °C and the developing solvent was HPLC-grade tetrahydrofuran (THF). As the molecular weight standard, single molecular weight polystyrene was used. The fabricated acrylic PSA films were characterized using Fourier transform infrared spectroscopy (FT-IR, Thermoscience, Nicolet 6700, Waltham, MA, USA). The characteristics and degree of curing reaction according to the monomer ratio were analyzed by the ratio of a hydroxyl group and a double bond peak. The glass transition temperature of acrylic PSA films was measured using a differential scanning calorimeter (DSC, PerkinElmer, DSC 8000), and the sample was measured under a nitrogen atmosphere from −50 to 200 °C at a temperature rising rate of 10 °C/min. Thermogravimetric analyzer (TGA7, PerkinElmer, Seoul, Korea) was used to confirm the thermal stability of the acrylic PSA film, and the samples were measured under a nitrogen atmosphere from 20 to 600 °C at a temperature rising rate of 10 °C/min.

### 2.5. Morphological and Rheological Analysis of Hydrophilic Acrylic PSA Films

Surface wettability according to the ratio of monomers was measured using a contact angle analyzer (SEO, Phoneix, AZ, USA), and the characteristics of the samples was compared through the angle of the droplet on the hydrophilic acrylic PSA film surface. The surface elements of hydrophilic acrylic PSA film were analyzed using X-ray photoelectron spectroscopy (XPS, Thermo Fisher, K-Alpha) with AlKa X-ray source. A hydrophilic acrylic PSA film measuring 25 mm × 200 mm was prepared and attached to a SUS substrate by 2–3 passes of the 2-kg-load rubber roller. After 30 min of attachment, peel strength was measured using 180° peel test at a tensile rate of 300 mm/min at room temperature. To confirm the orientation of polymer chain to the HEA content, the 1D X-ray diffraction (XRD, PANalytical, Empyrean, Malvern, UK) was measured and the experiment was conducted under the conditions of Cu-Ka X-ray source, 40 kV, and 30 mA. The scan range was 2θ = 2°–50° and was measured after attaching the hydrophilic acrylic PSA film to the Kapton film. To confirm the orientation of chains according to direction, the 2D XRD, wide-angle XRD (WAXS, Brucker, D8 discover, Billerica, MA, USA), was measured and the experiment was conducted under the conditions of Cu-Ka X-ray source, 40 kV, and 40 mA. The distance from the sample was maintained at 80 mm, and the range of 2θ = 2°–32° was measured. The viscoelastic behavior of the hydrophilic acrylic PSA film was analyzed using a rheometer (Anton paar, MCR 102, Graz, Austria) and measured from −40 to 100 °C under a strain of 0.4% and an oscillation frequency of 1 Hz.

### 2.6. Characterization of Affinity for Hydrophilic Materials and Biocompatibility

The hydrophilic materials PVA and HA were dissolved in D.I. water (solid content: 20 wt %) and coated on a PET film using a bar coater (Kipae, ComateTM 3000VH, Seoul, Korea). The coated hydrophilic films were prepared by drying at a temperature of 80 °C for 10 min on a hot plate. A hydrophilic acrylic PSA film measuring 25 mm × 200 mm was prepared. Then, the hydrophilic acrylic PSA film was attached to the fabricated hydrophilic film using 2–3 passes of the 2-kg-load rubber roller. After 10 min of attachment, peel strength was measured using T-type peel test at a tensile rate of 300 mm/min at room temperature. For the reliability of the evaluation method, commercial acrylic PSA, which has hydrophobic properties, were also evaluated for affinity with the hydrophilic film. A 3 wt % of isophorone diisocyanate was added to the commercial acrylic PSA, followed by uniform mixing using a Thinky mixer (Thinky, ARE-310, Laguna Hills, CA, USA). The mixed acrylic PSA was coated on a PET film and then dried for 10 min on a hot plate at 80 °C to prepare a hydrophobic acrylic PSA film. The fabricated acrylic PSA film was aged for one day in an oven at 70 °C. In addition, the fabricated 25 mm × 200 mm acrylic PSA was attached to the hydrophilic film using 2–3 passes of the 2-kg-load rubber roller. After 10 min of attachment, peel strength was measured using T-type peel test at a tensile rate of 300 mm/min at room temperature.

Cytotoxicity test was evaluated using the dead–live test using calcein-acetoxymethyl (AM) and propidium iodide (PI). After culturing the cells on the fabricated hydrophilic acrylic PSA film for two days, the toxicity was evaluated through the dead–live test of the cells. A confocal laser scanning microscope (Carl Zeiss, LSM-510, Oberkochen, Germany) was used for the evaluation. 

## 3. Results

### 3.1. Characterization of Synthesized Hydrophilic Acrylic Pre-Polymers and Acrylic PSA Films

**Scheme 1:**
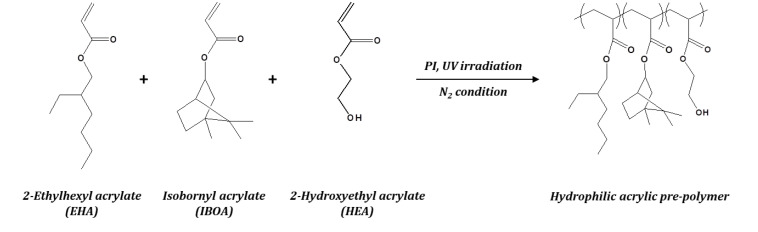
Synthesis of hydrophilic acrylic pre-polymers through photopolymerization.

As shown in Scheme 1 and Table 1, the acrylic pre-polymers were synthesized by controlling the contents of 2-ethylhexyl acrylate (EHA), isobornyl acrylate (IBOA), and 2-hydroxyethyl acrylate (HEA); especially, the characteristic change of the HEA content was analyzed. The monomer ratio and molecular weight of synthesized acrylic pre-polymers for the HEA content are shown in Table 1. It is confirmed that the molecular weight gradually increases from 504,000 (Acryl-2) to 880,000 g/mol (Acryl-40) with an increase in the HEA content. The molecular weight of the polymer is influenced by the polarity, volume, and hydrogen bonding of the solvent or used monomer [27,28]. When a polymer is polymerized through radical polymerization, backbiting occurs in the propagation step to form side branches in the polymer chain [29]. Here, the presence of hydrogen-bondable solvent such as alcohol or unreacted monomer with hydroxyl groups inhibited the backbiting phenomenon and increased the radical fraction at the end of the chain, leading to a higher polymerization speed and a higher molecular weight. Consequently, as the HEA content increased, backbiting was restricted by unreacted HEA and the molecular weight increased because the chain lengthened in a linear form. In addition, the addition of HEA affected the increase in molecular weight since it also improved the reactivity and reaction rate with alkyl acrylate [30]. Moreover, if the content of HEA was over 10 wt % (Acryl-10 to Acryl-40), the molecular weight tended to increase dramatically, as the reduction in the content of IBOA hard segment reduced the steric hindrance. 

The synthesized acrylic pre-polymers were analyzed by 1H-NMR, and the results are shown in Figure S2. In the acrylic pre-polymer, proton peaks of the side chains were identified at the same positions as the used monomers EHA, IBOA, and HEA, and they were broader than those of the monomers. The intensity of the peak was determined by the ratio of monomers, and the peak of the double bond was observed between 6 and 6.5 ppm for all acrylic pre-polymer. Through this, we confirmed that acrylic pre-polymers were successfully polymerized through UV irradiation with monomers’ ratio and obtained in a mixed form with unreacted monomers. Acrylic PSA films were prepared by coating pre-polymer and curing all residual monomers through secondary UV curing. In this case, it could not be analyzed by FT-NMR because it was not soluble in solvent. Therefore, the acrylic PSA films were characterized by FT-IR.

**Figure 1:**
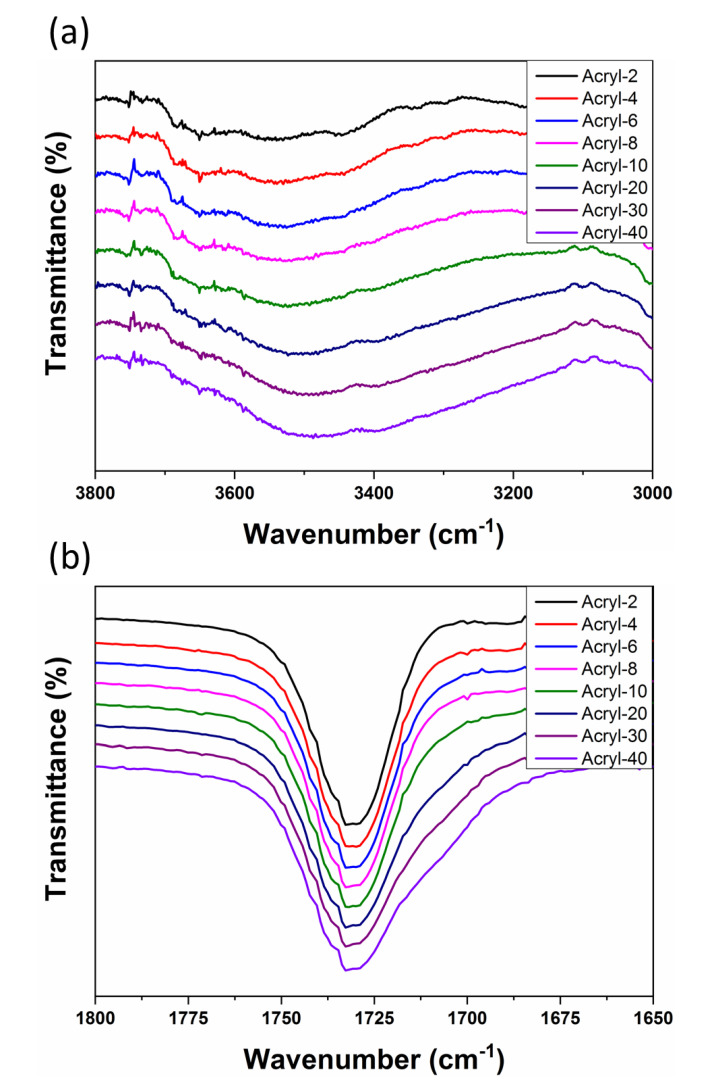
FT-IR spectrum of acrylic PSA films for HEA content: (**a**) O–H region; and (**b**) C=O region.

The FT-IR was measured for structural analysis of hydrophilic acrylic PSA film for HEA content, a hydrophilic segment. As shown in Figure S1, all acrylic PSA films did not show the vibration peak of the C=C double bond appearing in 1619–1637 cm^−1^, which can confirm that the polymer was successfully polymerized because of UV curing without any residual monomer. In addition, as the content of HEA increased from 2 to 40 wt %, the intensity of the O–H peak of the acrylic PSA film in 3446–3689 cm^−1^ increased and appeared to be broad. This is because hydrogen bonding increased with increasing O–H group content [31]. As shown in Figure 1a, the peaks of 3689 and 3652 cm^−1^ for Acryl-2 identified the trans and gauche forms of the O–H group that do not participate in the reaction, and the peaks of 3550 and 3446 cm^−1^ identified the hydrogen bond of C=O group and O–H group and the aggregate between O–H groups, respectively. In the case of Acryl-4 to Acryl-8, it can be shown that the peak of O–H groups was shifted to the short-wave region at 3550 and 3446 cm^−1^. This is because hydrogen bonding between polymer chains was strengthened by increasing HEA content. In the case of Acryl-10 to Acryl-40, the intensity of the peak of 3446 cm^−1^ drastically increased and broadened because the rate of aggregation of the O–H group was increased. As the HEA content increased from 10 to 40 wt %, the content of IBOA, a hard segment, decreased from 40 to 10 wt %. When the hard segment content decreased, the polymer chains became very flexible and the packing between the chains was induced more by hydrogen bonding, which was strengthened by the increase in HEA content. These results were also obtained in the peak of the C=O group, as shown in Figure 1b. The peak identified at 1730 cm^−1^ was shown for the C=O group to not participate in the reaction, which was confirmed to be the same in all acrylic PSA films without shift. The shoulder peak was identified at 1706 cm^−1^ from Acryl-10 to Acryl-40 because—as mentioned above—the packing characteristics between chains increased due to the flexible polymer chain. The more tightly packed chains facilitated the interaction between the O–H and C=O group, increasing the ratio of hydrogen bonding between the chains [32].

**Figure 2:**
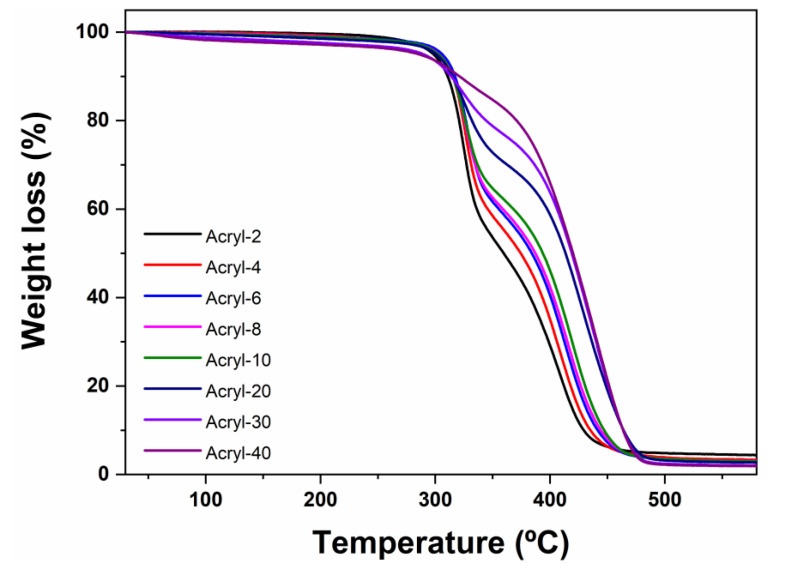
Thermal stability analysis of acrylic PSA films for HEA content.

The thermal properties of fabricated acrylic PSA film were analyzed by DSC and TGA; the glass transition temperature (*T*_g_) and thermal decomposition temperature (Td) are shown in Table 1 and Figure 2. In Table 1, the *T*_g_ from Acryl-2 to Acryl-8 increased from −9.50 °C to −6.86 °C, whereas, from Acryl-10 to Acryl-40, it decreased from −9.88 °C to −13.84 °C. In the former case, although the hard segment content was reduced, the increased HEA content strengthened the hydrogen bonding between the polymeric chains, thus inhibiting polymer mobility. Consequently, it was shown that the *T*_g_ of the polymer tended to increase [33,34,35]. Much more HEA was added in the latter case than in the former case; however, *T*_g_ still tended to decrease. This is because of the dramatic increase in chain flexibility with a decrease in hard segment content. Improvement in the flexibility of the chain reduced the *T*_g_ because the polymer chain mobility was easier. In conclusion, it was confirmed that hydrogen bonding at the ratio of HEA of less than 10 wt % had a greater effect on *T*_g_ than in the hard segment, whereas hard segment content had a greater effect on *T*_g_ than hydrogen bonding at a ratio greater than 10 wt %. Figure 2 shows the thermal decomposition temperature (*T*_d_) of hydrophilic acrylic PSA film. The temperature of acrylic PSA films was raised to 600 °C; all acrylic PSA films decomposed in three -steps [36,37]. The first weight loss occurred below 300 °C. At this stage, the weight loss occurred as the moisture, residual monomers, and hydrogen bonding decomposed. In the temperature range of 300–400 °C, the second weight loss of the acrylic PSA film appeared. The second weight loss was because the soft segment of the EHA and the hard segment of IBOA decomposed into acrylic acid. Since the hydrophilic segment of HEA was stable at high temperatures, the thermal stability also increased with an increase in the HEA content of the acrylic PSA film [38]. For this reason, Acryl-2 showed the greatest weight loss, while heat stability was improved by HEA in Acryl-40 to reduce weight loss. An increase in temperature above 400 °C resulted in a third weight loss, as the main chain of the acrylic polymer was decomposed at this step. From Acryl-2 to Acryl-10, there was no significant change in the *T*_d_ of the main chain, but, from Acryl-20, the *T*_d_ was slightly increased. This improvement in thermal characteristics was because of the increase in hydrogen bonding along with a decrease in the hard segment of IBOA [39]. In all acrylic PSA films, less than 5% char was formed and it was shown that the char formation decreased with an decrease in the content of the hard segment.

**Figure 3:**
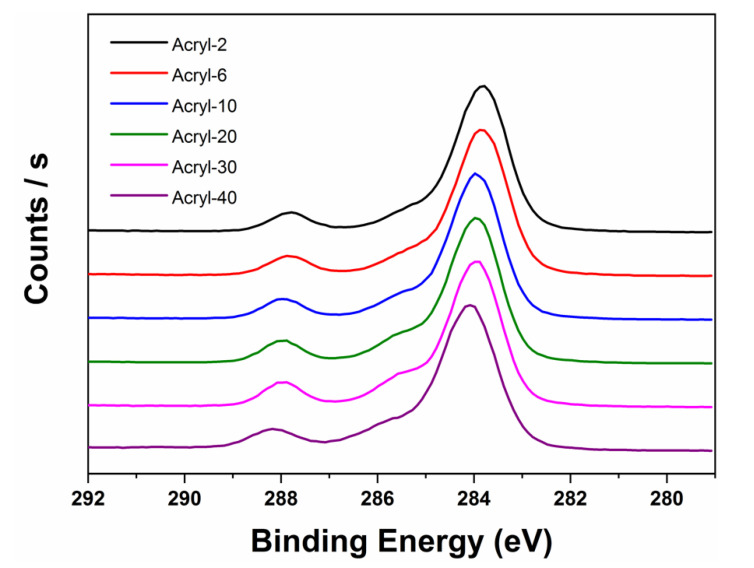
Surface element spectrum of acrylic PSA films for HEA content, carbon atom.

The XPS was measured for the elemental analysis of the acrylic PSA film surface for the HEA content and the results are shown in Figure 3 and Table 2. As shown in Figure 3, in all acrylic PSA films, the peaks of C‒C, C‒O, and C=O were identified at 283, 285, and 287 eV, respectively. From Acryl-2 to Acryl-8, with a content of less than 10 wt % of HEA, no significant peak shift or intensity changes were observed. On the other hand, from Acryl-10 to Acryl-40, with HEA content higher than 10 wt %, the peak shifted from 283.98 to 284.08 eV for C‒C. This is because hydrogen bonding was also strengthened because of the increased electronegativity of HEA. [40] The surface elements of the acrylic PSA film were analyzed using the ratio of binding energy and intensity of C‒C/C‒O peak and normalization was performed using the C‒C peak intensity. The increase in the ratio of C‒C/C‒O peak was considered as hydrophobicity and the decrease in the ratio was considered as hydrophilicity [41,42]. Acryl-2 and Acryl-6, with HEA content less than 10 wt %, slightly decreased the ratio of C–C/C–O from 5.09 to 4.84. Acryl-10 to Acryl-30, with an HEA content of more than 10 wt %, significantly decreased the ratio of C‒C/C‒O from 4.65 to 4.25. The surface hydrophilicity improved as HEA increased, and the proportion of the hydrophilic segment located on the surface also increased.

### 3.2. Morphological Analysis of Acrylic PSA Films

**Figure 4:**
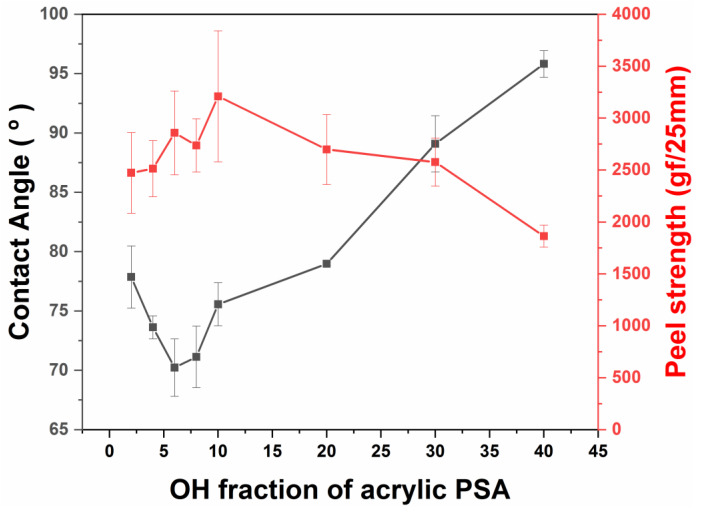
Surface contact angle and adhesive force analysis of acrylic PSA films for HEA content.

The contact angle was measured and analyzed to confirm the wettability change of the acrylic PSA for HEA. As shown in Figure 4, it was confirmed that the contact angle of acrylic PSA film decreased from 77.87° to 70.23° in the case of Acryl-2 to Acryl-8, thereby improving the hydrophilicity. This is because—as is widely known—more hydrophilic segments such hydroxyl groups were located on the acrylic PSA film surface as the HEA content increased. This is consistent with the results mentioned in Figure 3. As the proportion of the hydroxyl group on the surface increased, the interaction with water, which is polar, became stronger, resulting in increased surface wettability and improved hydrophilicity. However, the surface contact angle of Acryl-10 to Acryl-40 increased up to 92.29°, indicating hydrophobicity. These results are not consistent with those presented in Figure 3 and contradict the conventional theory that hydrophilicity improves as the hydrophilic group increases. The entanglement of the polymer chain can explain this phenomenon. [43] As shown in Table 1, when the HEA content was less than 10 wt %, the molecular weight was about 520,000 g/mol, but, when it was over 10 wt %, the molecular weight increased up to 880,000 g/mol. As the molecular weight of the polymer increased, the polymer chains became more entangled, which resulted in a decrease in free volume, thereby reducing the surface energy. Through this phenomenon, the surface hydrophobicity of the acrylic PSA film was also increased. Besides, the high molecular weight reduced the polymer–solvent affinity, and the surface hydrophobicity of acrylic PSA film was increased through these complex phenomena. As shown in Table 1, 10 wt % or higher HEA content increased the flexibility of the chain because of a decrease in the hard segment, and the free volume further decreased by the hydrogen bonding, strengthened by an excess hydroxyl group. Consequently, the hydrophobicity of the surface was further increased. In conclusion, Figure 3 and Figure 4 show that the surface properties of the acrylic PSA film had a greater effect on the entanglement of polymer chain by high molecular weight than the hydrophilic segment, such as a hydroxyl group, when HEA content was more than 10 wt %.

Figure 4 also shows the adhesive characteristics of the acrylic PSA film for the HEA content; the adhesion was evaluated through 180° peel test. All acrylic PSA films showed high adhesive force above 1800 gf/25 mm. When HEA increased from Acryl-2 to Acryl-10, the adhesion increased from 2474 to 3211 gf/25 mm, while Acryl-40, with the highest HEA content, had decreased adhesion of 1864 gf/25 mm. These results are because of the surface wettability of the acrylic PSA films. The adhesive characteristics of acrylic PSA film are greatly influenced by the wettability of the surface [44,45,46]. When the hydrophilicity of the acrylic PSA film is increased, the surface energy is increased to improve interaction with the substrate, thereby increasing the adhesive strength. On the other hand, increased hydrophobicity of acrylic PSA film reduces surface energy, thus reducing adhesion because of a lack of interaction with the substrate. These results are consistent with the surface wettability of the acrylic PSA film mentioned in Figure 4.

**Figure 5:**
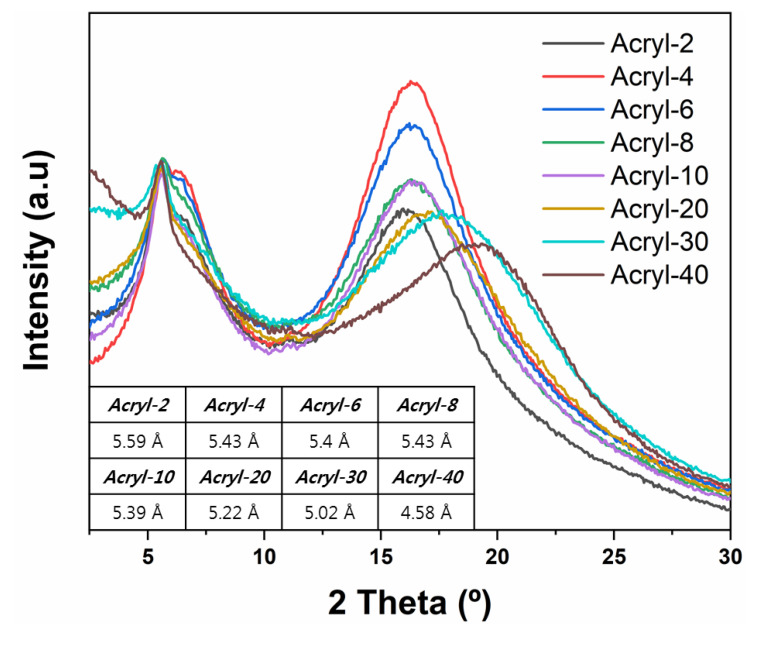

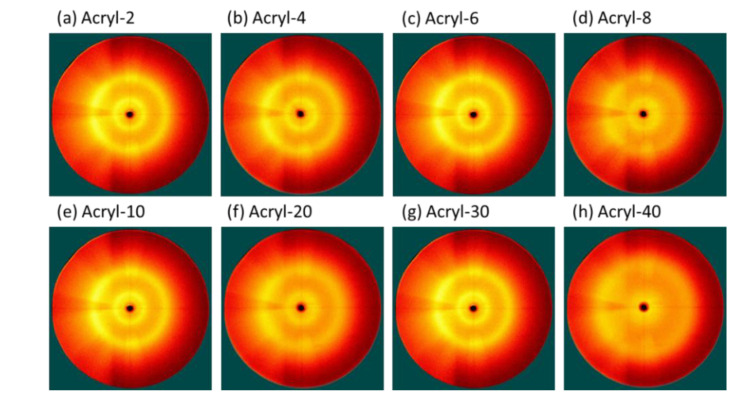
XRD pattern (up) and WAXS image (down) of acrylic PSA films for hydrophilicity.

Figure 5 exhibits the XRD investigation of acrylic PSA films to analyze the ordering structure of hydrophilicity. The acrylic PSA films were fabricated by UV curing after coating between the light and heavy release films. The distance between the polymer chains was calculated by Bragg’s equation and the results are shown in Figure 5 (up). Here, the peak of acrylic PSA films was red-shifted as the HEA content increased. The same peaks were observed at 5.62° in all acrylic PSA films, and, remarkably, noticeable changes in the peaks at 6.57° and 16.11° were observed with increased HEA content. Acryl-4 showed a drastic increase in the intensity of the two peaks at 6.57° and 16.11°, and it was confirmed that distance between the chains was closer when calculating the distance using Bragg’s equation, from 5.49 to 5.43 Å. This is because the proportion of hydrogen bonding increased. As the hydrogen bonding became stronger because of the increase in HEA content, the distance between chains became closer because of the improved packing characteristic [47,48,49,50]. Moreover, because of the high ratio of the hard segment, the polymer chain also had a hard characteristic, making it possible to arrange the chain in a relatively ordered form. The intensity of the peak at 16.11° also increased as a result of improved packing characteristics and relatively ordered polymer chains. In Acryl-6 and Acryl-8, the intensity of the peaks at 5.67° and 16.11° were reduced. This is because the IBOA content, a hard segment, decreased as the HEA content increased. Some reduction in hard segment content, compared to Acryl-4, resulted in a slight increase in chain flexibility, resulting in disordered orientation because of chain-to-chain hydrogen bonding [51,52,53]. From Acryl-10 to Acryl-40, with an HEA content of 10 wt % or more, the peak intensity of 6.57° was reduced; the peak of 16.35° became broader and markedly shifted to 19.35°. The distance between the polymer chains became remarkably close, from 5.43 to 4.58 Å, and amorphous properties increased with increasing HEA content. These results can support the reduction of free volume in the polymer chain mentioned in Figure 4. The reason behind this characteristic is that the polymer chain became more flexible as the IBOA content—a hard segment—decreased in proportion to a larger HEA content. Owing to a more flexible polymer chain and the strong hydrogen bonding, they exhibited disordered orientation. In conclusion, it was confirmed that the morphology of acrylic PSA film was significantly affected by the flexibility of the polymer chain and the strength of hydrogen bonding. Figure 5 (down) shows a WAXD image, which is a 2D XRD, with two ring forms appearing on all acrylic PSA films. Therefore, it was confirmed that the polymer chain of acrylic PSA was uniformly oriented in all directions.

### 3.3. Rheological Analysis of Acrylic PSA Films

**Figure 6:**
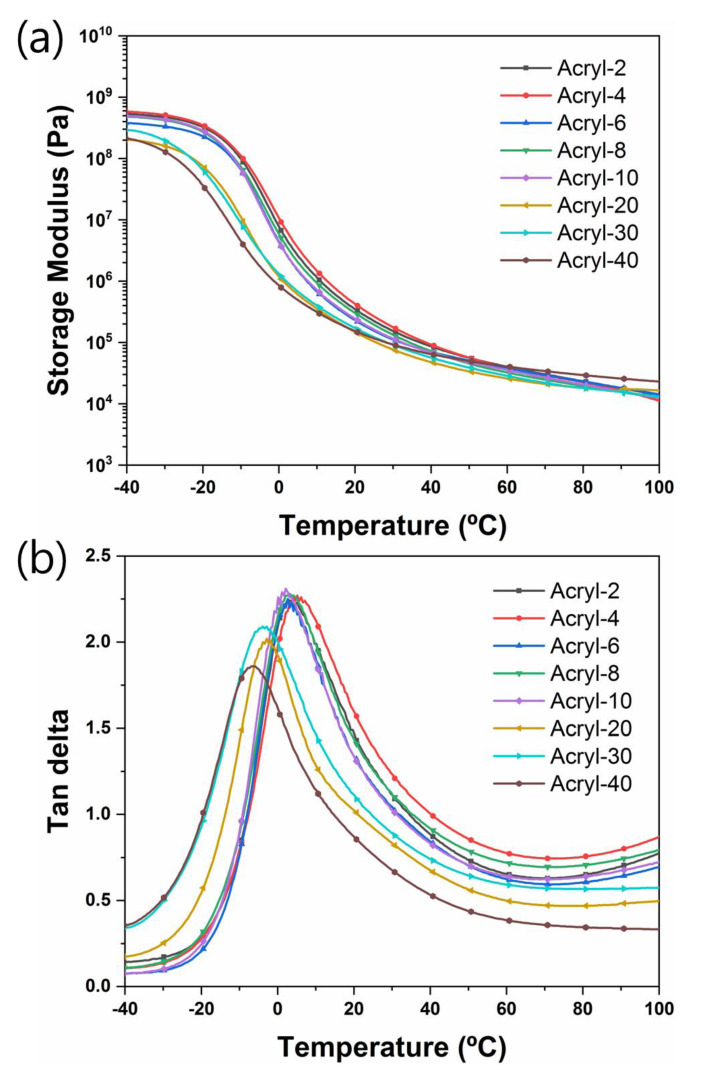
Modulus properties of acrylic PSA films: (**a**) storage modulus (G’); and (**b**) tan δ.

A rheometer was used to confirm the modulus change of the acrylic PSA films to the HEA content. The modulus was measured by changing the temperature from −40 to 100 °C at the same strain and frequency; the measured modulus is shown in Figure 6. As shown in Figure 6a, Acryl-2 to Acryl-8 showed a high modulus of 4.8 × 10^8^ to 5.8 × 10^8^ MPa in the low-temperature region at −40 °C. In addition, it was confirmed that the modulus of the medium temperature region, such as room temperature, tended to decrease as the HEA content increased. At the high-temperature region of 100 °C, there was no significant difference in modulus according to the HEA content, as in the low-temperature region. In the case of more than 10 wt %, e.g., Acryl-10 to Acryl-40, the modulus in the low-temperature region can be seen as decreasing drastically to 2 × 10^8^ MPa with an increase in the HEA content, which is considered to the result of the polymer chain’s flexibility. The increased HEA content results in a significant improvement in the flexibility of the polymer chain as the hard segment IBOA is reduced. Improved flexibility of the polymer chain also increased the flexibility of the acrylic PSA film, resulting in a decrease in its modulus. Additionally, it was found that the modulus of acrylic PSA film increased in the high-temperature region of 100 °C with an increase in HEA. These results can be attributed to hydrogen bonding, which became stronger with the increased HEA [32,48,54,55,56]. Compared with the results for less than 10 wt % HEA, e.g., Acryl-2 to Acryl-8, the proportion of hydrogen bonding was very high by the flexible chain for Acryl-40, and all hydrogen bonds were not decomposed, even at temperatures in excess of 100 °C. Therefore, no noticeable decrease in modulus was observed at high temperature, confirming that the modulus increased and flattened at high temperature.

### 3.4. Characterization of Affinity for Hydrophilic Materials and Biocompatibility

**Figure 7:**
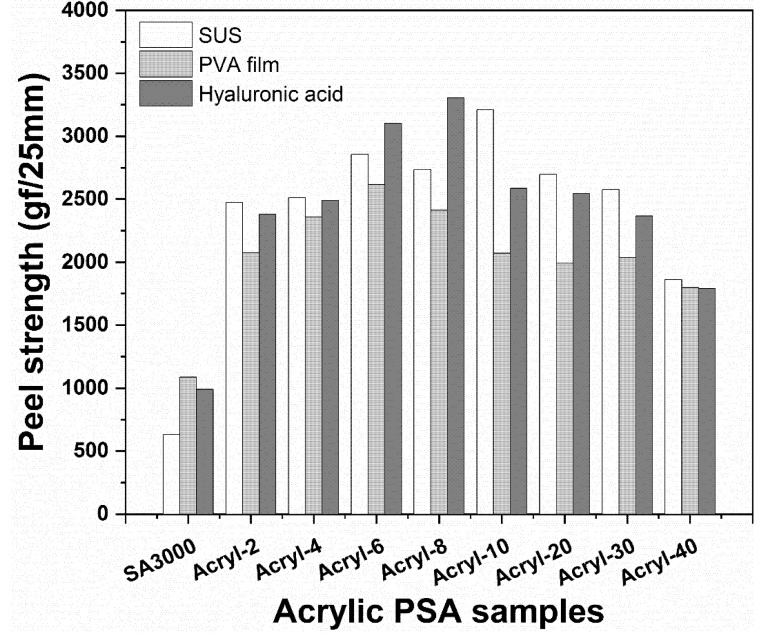
Evaluation of affinity of acrylic PSA films for hydrophilic materials: SUS, PVA film, and hyaluronic acid.

The affinity with hydrophilic materials for acrylic PSA films was evaluated, as shown in Figure 7. Poly(vinyl alcohol) (PVA) and hyaluronic acid (HA), hydrophilic materials with excellent biocompatibility, were selected and their affinity was evaluated according to the adhesion of the acrylic PSA film to hydrophilic materials. SA3000 material is a well-known pressure sensitive material based on acrylic derivatives with hydrophobic properties. Thus, we compared peel strength properties of synthesized polymers (hydrophilic) and SA3000 (hydrophobic) for hydrophilic substrates. The adhesion was measured by a T-type peel test with hydrophilic materials and synthesized acrylic PSA films. As shown in Figure 7, Acryl-6 and Acryl-8, which exhibited the maximum hydrophilic properties at 70.23° and 71.14°, showed high adhesion in PVA of 2413–2616 gf/25 mm, while in HA it exhibited a higher adhesion of 3106–3307 gf/25 mm. On the other hand, Acryl-40, which exhibited the maximum hydrophobic properties at 92.29°, exhibited the lowest adhesion at 1799 gf/25 mm in PVA and also at 1790 gf/25 mm in HA, similar to the adhesion in PVA. Consequently, it was confirmed that the affinity with hydrophilic materials was determined by the hydrophilicity of the acrylic PSA film. This is consistent with the results of the surface wettability of the acrylic PSA film in Figure 4. For comparison, the affinities between SA3000 hydrophobicity (Mw: 700,000–800,000 g/mol; contact angle: 95.19°) of commercial acrylic PSA and the hydrophilic materials were evaluated together. The commercial acrylic PSA exhibited relatively low adhesion of less than 1200 gf/25 mm regardless of the substrate because it is hydrophobic and cannot sufficiently react with the surface of hydrophilic materials such as PVA and HA. Interestingly, Acryl-40 exhibited similar hydrophobicity to commercial acrylic PSA but, as observed, combined more strongly with hydrophilic materials than commercial acrylic PSA. The hydrophobicity of Acryl-40 was the result of chain entanglement by high molecular weight. Figure 3 shows a relatively large distribution of hydrophilic segments such hydroxyl groups on the surface of the acrylic PSA film. These hydrophilic segments enable stronger secondary bonding with hydrophilic materials PVA and HA, resulting in higher adhesion than SA3000 of commercial acrylic PSA with similar hydrophobic properties. In conclusion, the affinity with hydrophilic materials was determined by the hydrophilicity of the acrylic PSA films, and it can be seen that the affinity could be easily and quickly evaluated through a T-type test. Thus, if we can control the peel strength for hydrophilic substrate by molecular structure based on acrylic derivatives, these PSA might be easily applied in various medical devices such as medical patch, needle patch etc.

The biocompatibility of acrylic PSA film for hydrophilicity was evaluated using in vitro cytotoxicity and skin irritation tests. After culturing the cells on the acrylic PSA film for two days, its cytotoxicity was evaluated through the cell’s survival using confocal microscopy. Toxicity was determined when the number of cultured cells on the acrylic PSA film was lower than the initial value; non-toxicity was determined when the number of the cultured cells was the same or higher than the initial value.

**Figure 8:**
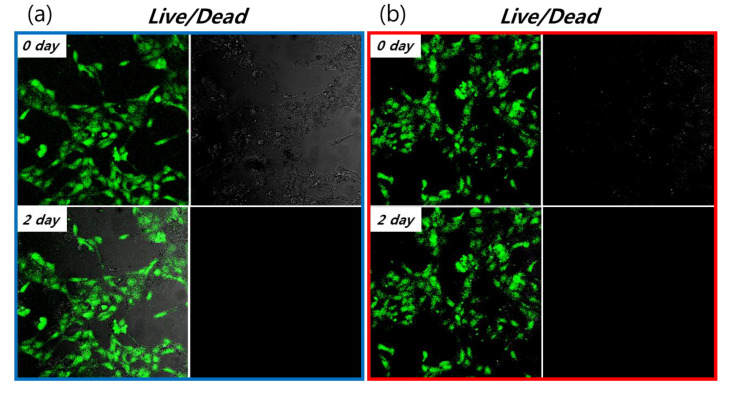
Live/dead confocal microscopy image of acrylic PSA films using calcein AM and propidium iodide: (**a**) Acryl-6, hydrophilic, and (**b**) Acryl-40, hydrophobic (live cell, green; dead cell, red).

The cytotoxicity test was performed by selecting the hydrophilic Acryl-6 and hydrophobic Acryl-40 in the acrylic PSA film, and the results are shown in Figure 8. As shown in Figure 8a, the number of cells for hydrophilic Acryl-6 remained the same, at the initial value, after two days. Likewise, the hydrophobic Acryl-40 in Figure 8b also exhibited the same results as Acryl-6, and it can be seen that the acrylic PSA film was non-toxic regardless of the hydrophilicity. These characteristics were considered because the residual monomer was cured through UV irradiation in the fabrication process of the acrylic PSA film, and no residual VOC was generated because a solvent was not used.

## 4. Conclusions

In this study, we synthesized acrylic PSAs through photo-polymerization and controlled their hydrophilicity using the content of 2-hydroxyethyl acrylate (HEA). The molecular weight of acrylic PSAs gradually increased from 504,000 (Acryl-2) to 880,000 g/mol (Acryl-40) with an increase in the HEA content. It can be seen that the surface hydrophilicity improved as HEA increased, and the proportion of the hydrophilic segment located on the surface also increased. However, the surface contact angle of acrylic PSA film over HEA 10 wt % increased up to 92.29°, indicating hydrophobicity. In conclusion, it can be confirmed that the surface properties of the acrylic PSA film have a greater effect on the entanglement of polymer chain by high molecular weight than the hydrophilic segment when HEA content is more than 10 wt %. Synthetic acrylic PSAs exhibited amorphous property and it was confirmed that the morphology of acrylic PSA film was significantly affected by the flexibility of the polymer chain and the hydrogen bonding. In addition, we investigated a simple method for evaluating affinity with acrylic PSAs and hydrophilic materials using the T-type peel test. Hydrophilic acrylic PSA films showed biocompatibility through cytotoxicity test, confirming that synthesized hydrophilic acrylic PSA films show potential for medical applications.

## Figures and Tables

**Table 1:** Monomer composition and molecular weight of hydrophilic acrylic pre-polymers.

Acrylic Syrups	EHA ^a^	IBOA ^b^	HEA ^c^	Characterization				
				*M*_n_ (g/mol)	*M*_w_ (g/mol)	PDI	*T*_g_^d^ (°C)	*T*_d_^e^ (°C)
Acryl-2	50	48	2	441,000	568,000	1.29	−9.50	298
Acryl-4		46	4	335,000	478,000	1.43	−9.15	303
Acryl-6		44	6	328,000	504,000	1.53	−9.11	306
Acryl-8		42	8	331,000	518,000	1.56	−6.86	303
Acryl-10		40	10	312,000	645,000	2.07	−9.88	302
Acryl-20		30	20	382,000	763,000	1.99	−10.98	301
Acryl-30		20	30	374,000	788,000	2.11	−12.77	289
Acryl-40		10	40	616,000	880,000	1.43	−13.84	286

^a^ 2-Ethylhexyl acrylate; ^b^ isobornyl acrylate; ^c^ 2-hydroxyethyl acrylate; ^d^ measured by DSC; ^e^ measured by TGA, defining the 5 wt % weight reduction point as Td.

**Table 2:** Measurement of surface element and binding energy acrylic PSA films for HEA content.

Sample	Binding Energy (eV)				
	E_C-C_	Intensity	E_C-O_	Intensity	E_C-C_/E_C-O_
Acryl-2	283.78	67,200	285.28	13,200	5.09
Acryl-6	283.78	66,900	285.28	13,600	4.92
Acryl-10	283.98	67,300	285.38	13,900	4.84
Acryl-20	283.98	66,900	285.48	14,400	4.65
Acryl-30	283.98	66,800	285.58	15,700	4.25
Acryl-40	284.08	67,000	285.58	15,500	4.32

## Supplementary Materials

The following are available online at https://www.mdpi.com/2073-4360/12/7/1504/s1, Figure S1: FT-IR spectrum of acrylic PSA films for HEA content: total region, Figure S2: ^1^H-NMR spectrum of acrylic pre-polymers for HEA content.
