# Supplementary material for: Change of Characterization and Film Morphology Based on Acrylic Pressure Sensitive Adhesives by Hydrophilic Derivative Ratio
Source: Polymers (Basel). 2020 Jul 7;12(7):1504. doi: 10.3390/polym12071504 (PMC7408043; doi:10.3390/polym12071504)
Supplement: Supplementary file 1 [file polymers-12-01504-s001.pdf]

Correlation between Surface Wettability and Orientation of Acrylic Pressure Sensitive Adhesives by Hydrophilic Derivative Ratio

Woong Cheol Seok [1, 2], Jong Tae Leem [1, 2], Ju Hui Kang [1], Young Jun Kim[2], Sangkug Lee[1] and Ho Jun Song [1,*]

[1] Green and Sustainable Materials R&D Department/Research Institute of Clean Manufacturing System, Korea Institute of Industrial Technology, 89 Yangdaegiro-gil, Ipjang-myeon, Seobuk-gu, Cheonan-si, Chungcheongnam-do, 331-822, Republic of Korea

[2] School of Chemical Engineering, Sungkyunkwan University,2066, Seobu-ro Jangan-gu, Suwon-si, Gyeonggi-do, 440-746, Republic of Korea

Phone : 82-41-589-8467; Fax: 82-41-589-8550; E-mail: song3026@kitech.re.kr*

*Corresponding Author

Ph. D. Ho Jun Song, E-mail : song3026@kitech.re.kr

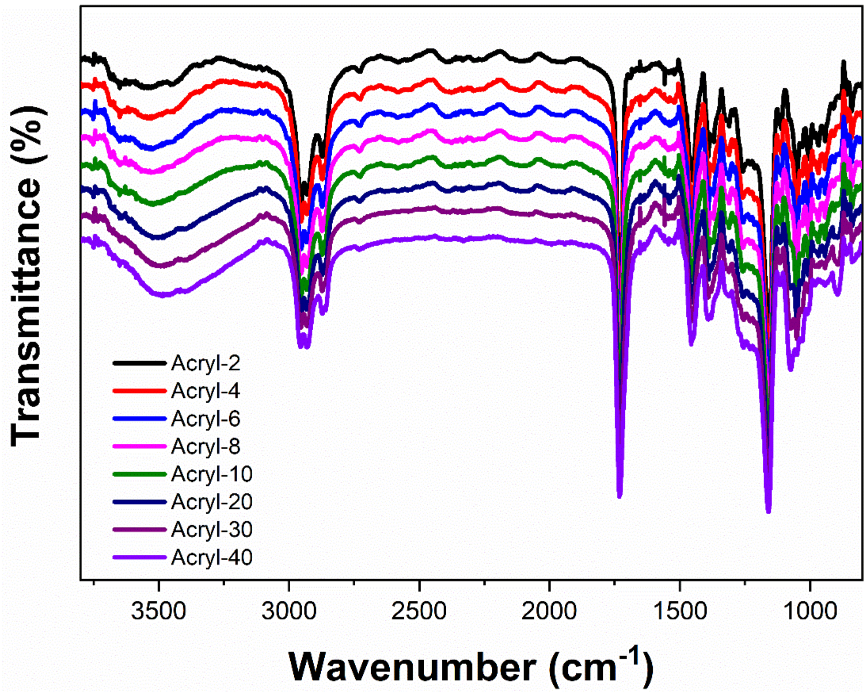

Figure S1. FT-IR spectrum of acrylic PSA films for HEA content; total region.

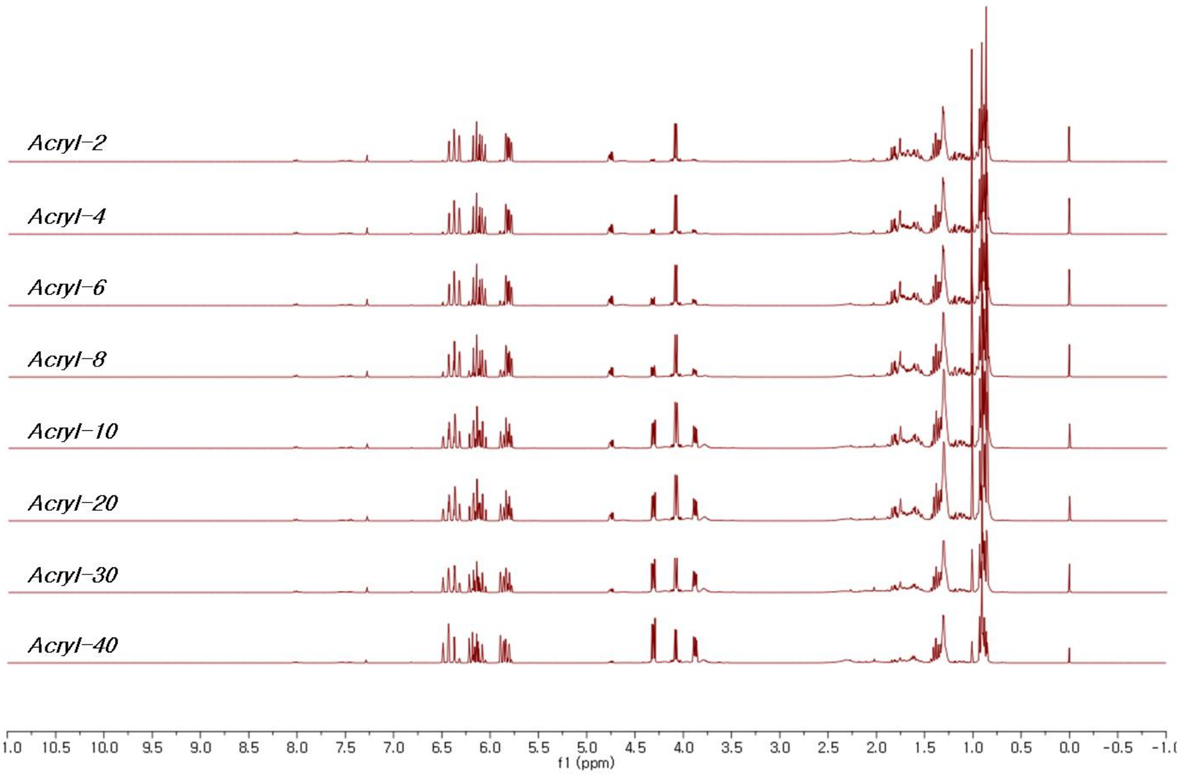

Figure S2. ^{1}H-NMR spectrum of acrylic pre-polymers for HEA content.
